# Combined Metabolomic and NIRS Analyses Reveal Biochemical and Metabolite Changes in Goat Milk Kefir under Different Heat Treatments and Fermentation Times

**DOI:** 10.3390/biom14070816

**Published:** 2024-07-09

**Authors:** Rubén Sánchez-Rodríguez, Carlos Terriente-Palacios, Juan García-Olmo, Sonia Osorio, Manuel J. Rodríguez-Ortega

**Affiliations:** 1 Departamento de Bioquímica y Biología Molecular, Universidad de Córdoba, Campus de Excelencia Internacional CeiA3, 14071 Córdoba, Spain; biorsrodriguez@gmail.com; 2 Institute for Mediterranean and Subtropical Horticulture “La Mayora”, Department of Molecular Biology and Biochemistry, University of Málaga-Consejo Superior de Investigaciones Científicas (IHSM-CSIC-UMA), 29071 Málaga, Spain; cterrient@uma.es (C.T.-P.); sosorio@uma.es (S.O.); 3 Servicio Central de Apoyo a la Investigación, Universidad de Córdoba, Campus de Excelencia Internacional CeiA3, 14071 Córdoba, Spain; nir@uco.es

**Keywords:** fermented milk, metabolome, metabolites, near-infrared spectroscopy, sample classification

## Abstract

Dairy products are an important source of protein and other nutrients in the Mediterranean diet. In these countries, the most common sources of milk for producing dairy products are cow, goat, sheep, and buffalo. Andalusia is traditionally the largest producer of goat milk in Spain. Kefir is a fermented product made from bacteria and yeasts and has health benefits beyond its nutritional properties. There is a lack of knowledge about the molecular mechanisms and metabolites that bring about these benefits. In this work, the combination of analytical techniques (GC-FID, UHPLC-MS-QToF, GC-QqQ-MS, and GC-ToF-MS) resulted in the detection of 105 metabolites in kefir produced with goat milk from two different thermal treatments (raw and pasteurized) fermented at four time points (12, 24, 36, and 48 h, using 0 h as the control). Of these, 27 metabolites differed between kefir produced with raw and pasteurized milk. These changes could possibly be caused by the effect of pasteurization on the microbial population in the starting milk. Some interesting molecules were identified, such as shikimic acid, dehydroabietic acid, GABA, and tyramine, which could be related to antibacterial properties, strengthening of the immune system, and arterial pressure. Moreover, a viability assay of the NIRS technique was performed to evaluate its use in monitoring the fermentation and classification of samples, which resulted in a 90% accuracy in comparison to correctly classified samples according to their fermentation time. This study represents the most comprehensive metabolomic analysis of goat milk kefir so far, revealing the intricate changes in metabolites during fermentation and the impact of milk treatment.

## 1. Introduction

The consumption of goat milk and its derived products holds significant importance in Mediterranean countries. Goat milk is very important in the renowned Mediterranean diet, valued for its nutritional richness and cultural significance over centuries [1]. Spain is one of the largest producers of goat livestock worldwide, and the use of goat milk and products like cheese is deeply rooted in the country’s culinary traditions. The high protein and mineral contents of goat milk make it a valuable source of nutrients for the population, contributing to its importance in Spain’s agricultural and dietary landscape [2].

Kefir, a dairy product resulting from acid-alcoholic fermentation, has been increasingly recognized for its potential health benefits [3,4]. The consumption of this fermented milk has been associated with improved digestion, modulation of the immune system, and potential anti-inflammatory properties [5]. This has led to a growing interest in milk kefir as a functional food in Spain and worldwide. In particular, goat milk kefir is gaining attention due to the unique properties of goat milk and the potential synergistic effects of kefir fermentation on its nutritional value and health benefits [6]. While cow milk kefir has been extensively studied, goat milk kefir remains less explored despite goat milk’s nutritional advantages, such as its easier digestibility, lower allergenicity, and favorable lipid profile when compared to cow milk [7,8].

Research using omics approaches has made significant contributions to understanding the molecular features of milk kefir. Several studies have determined the peptide composition of cow milk kefir, which has been the first and most characterized so far [9,10,11,12]. More recently, studies performed by our research group have characterized goat and sheep milk kefir at different fermentation times using a peptidomics approach, shedding light on the dynamic changes in protein digestion during fermentation and identifying potential peptides with biological activity [13,14]. Another recent study highlighted the intricate metabolic interactions and microbial community diversity of kefir grains, emphasizing the role of metabolomics in unraveling the complex metabolic processes underlying cow milk kefir fermentation [15]. Metabolomic analysis can play a crucial role in the biochemical characterization of kefir and other dairy products. By studying the complete set of small-molecule metabolites present in kefir, metabolomics may provide a comprehensive understanding of the metabolic processes occurring during fermentation and their impact on the properties of the final product. Also, metabolomic approaches enable the identification of bioactive compounds, flavor precursors, and metabolic pathways associated with the beneficial properties of foods, including kefir. This information can be highly valuable for quality control, product development, and understanding the potential health effects of kefir consumption [16].

This study focuses on the metabolomic analysis of goat milk kefir, aiming to characterize the differences between raw and pasteurized goat milk kefir throughout various fermentation times. For this, we have performed a comprehensive range of analytical techniques to characterize the metabolome in different conditions, as well as the near-infrared spectroscopy (NIRS) technique to better characterize and discriminate samples. Our metabolomics approach, which, to our knowledge, is the first performed on this product, offers a comprehensive way to understand the biochemical changes during fermentation, providing insights into the nutritional and therapeutic potential of goat milk kefir.

## 2. Materials and Methods

### 2.1. Reagents

Methanol (hypergrade for LC-MS, LiChrosolv^®^), chloroform (for analysis, EMPARTA^®^), water (hypergrade for LC-MS, LiChrosolv^®^), and pyridine (for analysis, EMSURE^®^) were from Supelco (Merck KGaA, Darmstadt, Germany). Sigma-Aldrich Chemie (Sant Quentin Fallavier, France) supplied ribitol, methoxyamine hydrochloride (purity 98%), and pure standards for the target compounds. *N*-methyl-*N*-trimethylsilyl-trifluoroacetamide (MSTFA) was from Macherey-Nagel (Düren, Germany).

### 2.2. Kefir Production

Kefir grains (Kefiralia, Gipuzkoa, Spain) were used to ferment goat milk, both raw and pasteurized at 74 °C, provided by a local farm (Quesería “Los Peña”, Baena, Córdoba, Spain). Prior to the definite samples, the kefir grains were conditioned in the two types of milk (raw and pasteurized) for one week, adding the grains (5% *w*/*v*) to both types of milk, i.e., raw and pasteurized. After that, samples were prepared by adding kefir grains (5% *w*/*v*) to either raw or pasteurized goat milk, at four different times (after 12, 24, 36, and 48 h of fermentation), using the corresponding non-fermented milk (time 0) as the control. Fermentations were performed in an incubator at 25 °C under aerobic conditions and without shaking. Three biological replicates were made for each type of sample. The fermented milk, known as kefir, was separated from the grains using a sieve, and then the coagulated caseins and remaining microbial cells were removed by centrifugation at 4500× *g* for 10 min at 4 °C. The samples were stored at −20 °C in 50 mL conical centrifuge tubes until further metabolomics or NIRS analysis. Aliquots of non-fermented raw and pasteurized milk were also frozen to be subject to the same treatment as the fermented samples.

### 2.3. Analysis of Fatty Acids by GC-FID

Cold extraction of fats from kefir samples was carried out according to a previously described method [17]. The resulting lipid fraction was subsequently subjected to a methylation reaction with 10% (*w*/*v*) KOH in methanol using a vortex. After decanting, a small volume of the supernatant phase, containing the methyl esters, was taken in a vial. Gas chromatography equipment with a GC-FID Clarus 500 flame ionization detector (PerkinElmer^®^, Waltham, MA, USA) was used for fatty acid analysis. The capillary column used was a BPX70 (SGE Analytical Science^TM^, Melbourne, Australia), specific for methyl esters from fatty acids. Hydrogen was used as the carrier gas at a pressure of 25 PSI. The injector and detector temperatures were set at 235 and 250 °C, respectively. For each analysis, 1 μL of the sample was injected in split mode. The column oven temperature was maintained at 170 °C for the first 10 min, followed by a 5 min ramp to 235 °C, which was kept stable for an additional 3 min. Data acquisition and processing were performed using Total Chrome Navigator software (PerkinElmer^®^, Waltham, MA, USA). The fatty acid profile was expressed as a % relative area of each peak recorded.

### 2.4. Analysis of Volatile Compounds by Headspace GC-QqQ-MS

For the analysis of volatile compounds with this technique, 1 g of kefir sample was weighed and 5 μL of a 100 ppm internal standard solution was added to a 10 mL vial. The analysis was carried out on a gas chromatographer coupled to a Scion TQ triple quadrupole GC-MS (QqQ) mass detector (Bruker Daltonics, Bremen, Germany). The GC was equipped with a DB-5MS capillary column (30 m in length, 0.25 mm in inner diameter, 0.25 μm in phase thickness, Agilent, Santa Clara, CA, USA). Incubation was performed at 80 °C for 12 min and 0.5 mL was injected from the headspace of the vial. The injector temperature was set at 225 °C and a 1–10 split. For the separation of the compounds, the following temperature program was set for the oven: 40 °C for the first 2 min, followed by the first temperature ramp of 10 °C/min until 175 °C was reached, and the second ramp of 35 °C/min until 275 °C was reached. The analyzer was operated in full-scan mode in the *m*/*z* range from 33 to 235. The source and transfer line temperatures were set at 250 °C. The identification of each compound was carried out by comparison of its experimental mass spectra with those of the NIST library (https://chemdata.nist.gov, accessed on 2 December 2023).

### 2.5. Analysis of Non-Volatile Compounds by GC-ToF-MS

Kefir samples were processed as previously described [18]. A ribitol solution (0.2 mg/mL in ultrapure water) was used as an internal quantitative standard. Briefly, approximately 80 mg of the fresh sample was extracted with 480 µL of a methanol–ribitol solution (4% *v*/*v*) and vortexed. Samples were shaken at 70 °C for 15 min and centrifuged at 14,000 rpm for 10 min (microcentrifuge 5415 R, Eppendorf AG, Hamburg, Germany). Supernatants were transferred to new 2 mL Eppendorf tubes and resuspended in 250 µL of chloroform and 500 µL of ultrapure water. After vortexing and centrifugation at 4000 rpm for 15 min, 150 µL from the upper phase was dried for at least 3 h without heating. The dried extracts were derivatized by using 40 µL of methoxyamine hydrochloride in pyridine (20 mg/mL, *p*/*v*) and shaken at 37 °C for 2 h. After adding 70 µL of the MSTFA–Fatty acid methyl ester solution (20% *v*/*v*) (*N*-Methyl-*N*-trimethylsilyl-trifluoroacetamide), samples were shaken at 37 °C for 30 min and transferred into GC vials. GC-TOF-MS analysis was performed using an Agilent 7890B GC system (Agilent Technologies, Palo Alto, CA, USA) with a Gerstel Multipurpose MPS autosampler (GERSTEL GmbH & Co. KG, Pforzheim, Germany) and a Pegasus HT TOF-MS (Leco Corporation, St. Joseph, MI, USA). The analytical program employed for sample analysis was adopted from a previous study [18]. Three biological replicates for each sample were injected and the analyses were performed in random order to reduce the bias. The results for each metabolite were expressed using a relative index (RI), which was calculated according to the following equation:

$$RI=\frac{{Peak\;area}_{metabolite}/{Peak\;area}_{Internal\;standard}}{Sample\;weight}$$

### 2.6. Analysis of Non-Volatile Compounds by UHPLC-QToF-MS

Liquid–liquid pre-extraction was performed, in which 1 g of the kefir sample was mixed with 10 mL of methanol. Also, 100 μL of a 10 ppm internal standard solution was added to compensate for systematic and random errors throughout the process. From the supernatant resulting from the extraction, a small fraction was taken, filtered to remove any remaining protein in suspension, and transferred to a vial for analysis. The analysis of polar metabolites was performed using high-pressure liquid chromatograph equipment coupled to a G2XS quadrupole-time-of-flight (QToF) UHPLC-MS analyzer (Waters^TM^, Milford, MA, USA). The analyzer conditions, in positive electrospray ionization and sensitivity mode, were as follows: capillary and cone voltages of 0.8 and 20 kV, respectively; mass acquisition range (*m*/*z*) of 60–1200 Da; cone gas and desolvation gas flow rates of 50 and 1000 L/h, respectively; and source and desolvation gas temperatures of 120 and 500 °C. The QToF was operated in MSE mode with a low collision energy of 6 eV and a high energy ramp from 10 to 45 eV. For the acquisition, a 200 ppb solution of leucine (in acetonitrile/water 0.1% formic acid, 50:50), infused at 10 s intervals for internal mass correction, and a 0.5 mM solution of sodium formate (in 2-propanol/water, 90:10) were used as a reference and a 0.5 mM solution of sodium formate (in 2-propanol/water, 90:10) was used as a calibrant. An ACQUITY UPLC^®^BEH C18 column (2.1 × 50 mm, 1.7 μm) (Waters^TM^, Milford, MA, USA) at 40 °C was used for metabolite separation. The mobile phase flow rate was 0.4 mL/min, using two mobile phases: A (0.1% formic acid) and B (methanol). An elution gradient of 18 min duration was used: 0–11 min 4% B: 11–13.8 min, 100%: 13.8–14 min, 100%: 14–18 min, 96%. In all cases, the volume of the sample injected was 2 μL. Data acquisition and processing were performed using the software UNIFI^TM^ v1.8.0 (Waters^TM^, Milford, MA, USA). Tentative identification of polar metabolites was performed by *m*/*z* search, based on previously published work [19]. The results were expressed in relative areas based on the internal standard used.

### 2.7. NIRS Analysis

The kefir samples were scanned by reflectance on a Foss DS2500 NIRS spectrophotometer (Foss Analytics, Hilleroed, Denmark). The equipment monitors the VIS (visible) region between 400 and 1100 nm as well as the NIR (near infrared) region between 1100 and 2500 nm. Therefore, the monitored region encompasses the entire region of the electromagnetic spectrum between 400 nm and 2500 nm. Next, 750 µL of each sample was deposited in the transport module with a circular gold-coated aluminum lid to promote the correct distribution of the kefir on the quartz glass and to avoid the presence of bubbles. The collected spectra were taken every 8.5 nm, and the absorbance data were obtained as log(1/R), where R corresponds to the reflectance. Two spectra were obtained for each sample, giving a total of 60 spectra corresponding to 30 for each type of milk (raw and pasteurized) and 6 for each fermentation time (0, 12, 24, 36, and 48 h).

### 2.8. Data and Statistical Analysis

The software used for the analysis was RStudio version 2023.3.1.44619. The principal component analysis (PCA) and discriminant analysis of principal components (DAPC) were performed using relative area data or NIRS spectra using the following packages: stats, ggplot2, pls, FactoMiner, factoextra, MASS, tidymodels, and caret. The NIRS spectra were previously normalized by SNV (Standard Normal Variate) and MSC (Multiple Scatter Correction), where MSC was chosen as the best normalization. The regions of the electromagnetic spectra used comprise the whole VIS + NIR region. The DAPC was run considering 15 PCs as the optimal number of PCs to retain. The confusion matrices of the classification models were obtained using Leave One Out Cross Validation (LOOCV). The statistical significance of differences was determined by one-way ANOVA with Tukey’s HSD post-hoc test (*p* < 0.05) considering the following *p*-adjusted values: ns, *p* > 0.05; *, *p* < 0.05; **, *p* < 0.01; and ***, *p* < 0.001. Heatmaps were generated using the ComplexHeatmap and circlize packages. To represent metabolite data with a relative area equal to 0, a value of 0.01 was added to the entire data matrix. The log_2_(Fold change) was then calculated and used to generate the heatmaps. The line plots were generated using ggplot2 and ggprism.

## 3. Results

### 3.1. Metabolomic Analysis of Goat Milk Kefir

With the aim of characterizing the qualitative and quantitative changes of metabolites during the fermentation time and the differences between raw and pasteurized milk in the production of kefir, we applied a range of analytical techniques to identify fatty acids (GC-FID) and volatile (GC-QqQ-MS) and non-volatile compounds (UHPLC-ToF-MS, GC-ToF-MS). In total, we found 105 unique compounds in all the different categories of samples—two thermal treatments, i.e., raw and pasteurized milk, and five fermentation times, i.e., 0, 12, 24, 36, and 48 h—whose identities and the technique with which they were identified are shown in Table 1. Out of the 105 metabolites, 21 were fatty acids identified by GC-FID; 26 volatile compounds were detected by GC-QqQ-MS; 48 other molecules were found by GC-ToF-MS; and 15 compounds were identified using UHPLC-ToF-MS. Five compounds were overlapping between these two latter techniques.

**Figure 1:**
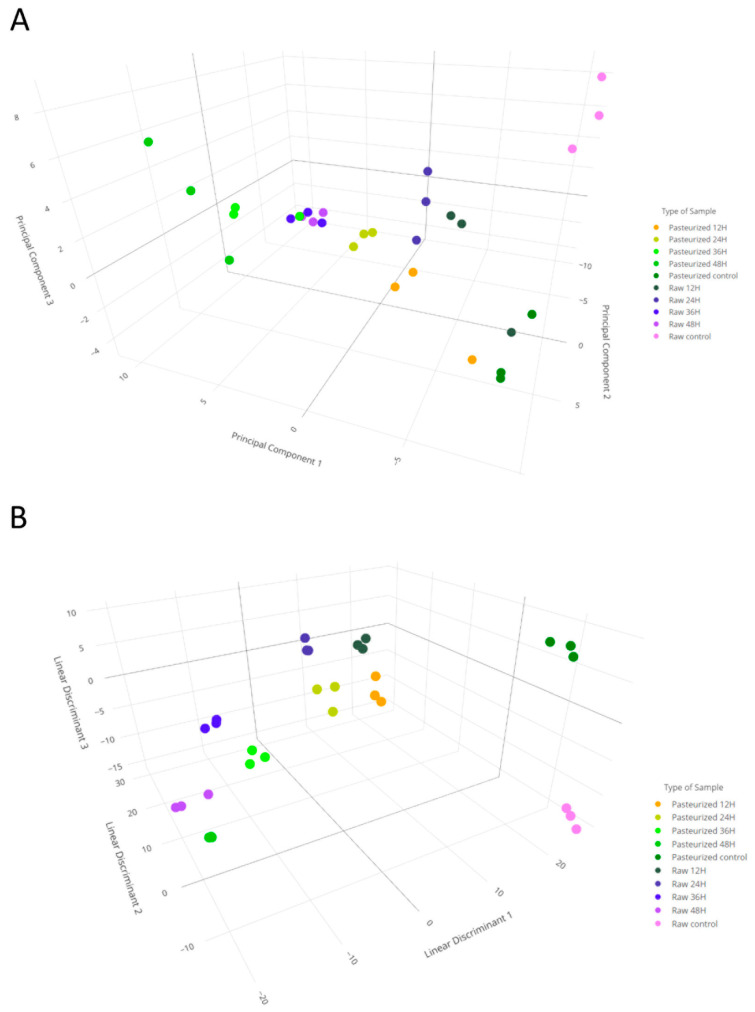
Score plots of three-dimensional principal component analysis (PCA, panel **A**) and discriminant analysis of principal components (DAPC, panel **B**) of all the metabolites identified using different metabolomics platforms in goat milk kefir.

The three-dimensional principal component analysis (PCA) using all the metabolites detected with the above-cited techniques explained 67.02% of the variance (40.41% for PC1, 16.91% for PC2, and 9.7% for PC3), clearly separating the two non-fermented types of milk (raw and pasteurized), but confounding some fermentation times, especially those at 36 and 48 h (Figure 1A). However, the discriminant analysis of principal components (DAPC) clearly separated the 10 different types of samples (Figure 1B), both according to the heat treatment of milk (raw vs. pasteurized) and the fermentation time (0, 12, 24, 36, and 48 h).

**Figure 2:**
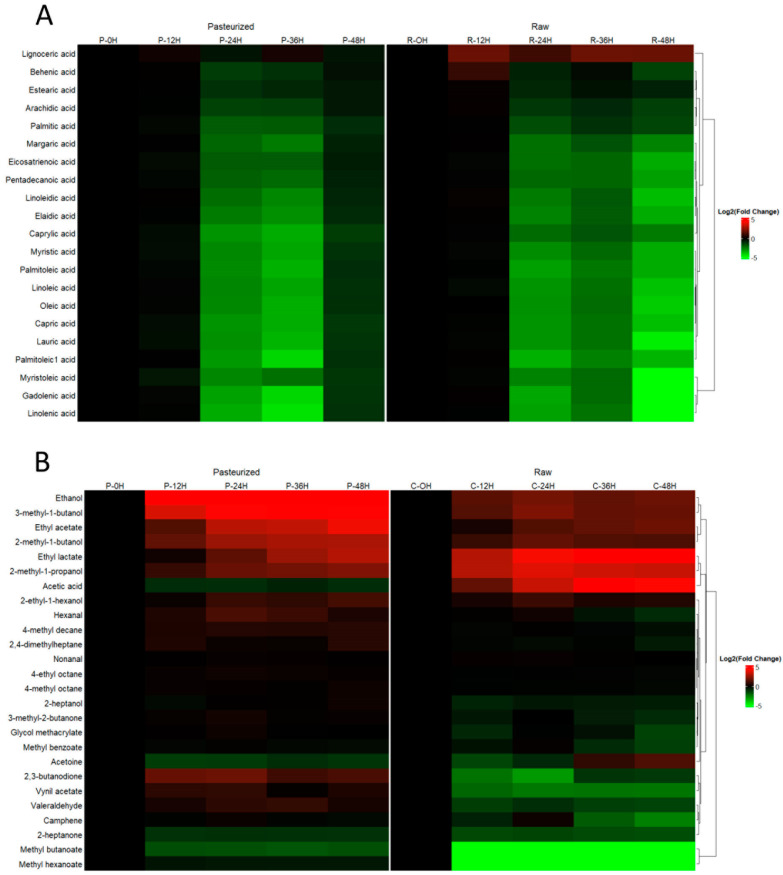

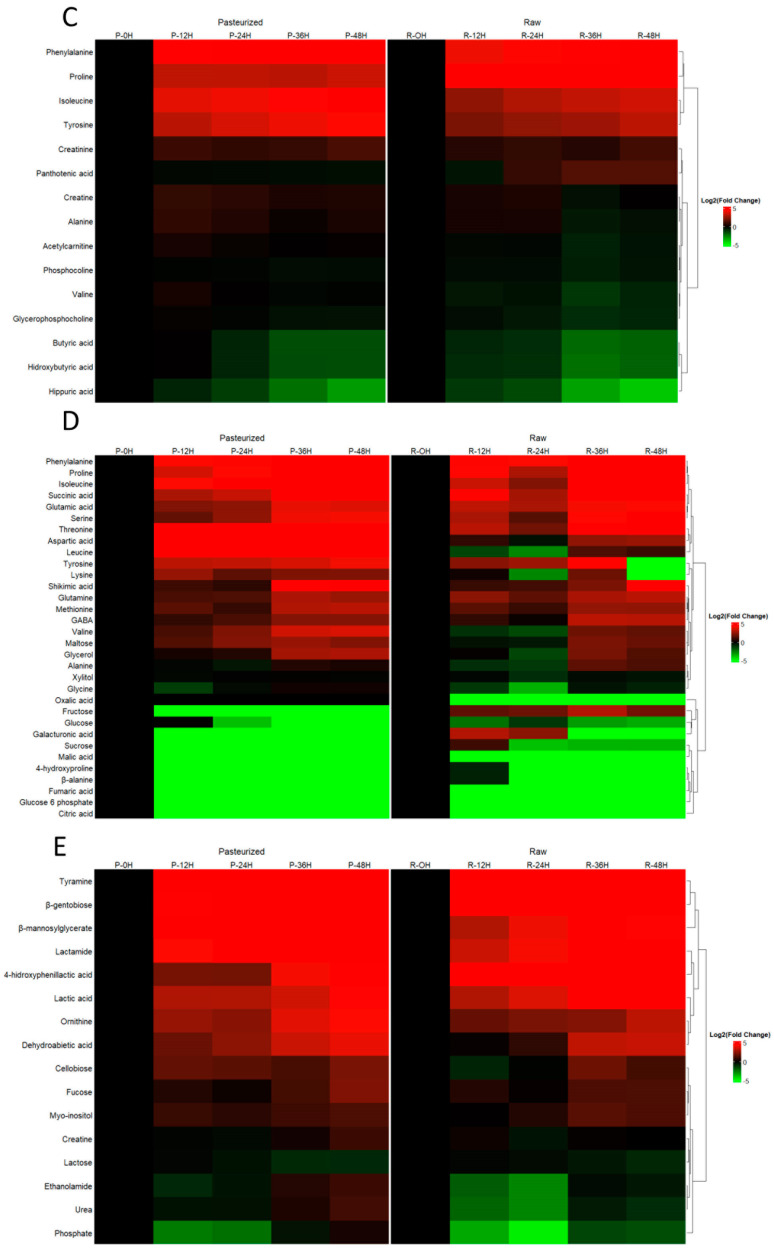
Heatmap visualizations of differences in metabolites identified in goat milk kefir with the different analytical procedures: (**A**) GC-FID for fatty acids; (**B**) Headspace GC-QqQ-MS for volatile compounds; (**C**) UHPLC-QToF-MS for non-volatile compounds using commercial standards for identification; (**D**) GC-ToF-MS for non-volatile compounds using commercial standards for identification; and (**E**) GC-ToF-MS for non-volatile compounds using spectra libraries for identification.

Figure 2 shows heatmaps and hierarchy cluster analyses of the found metabolites grouped according to the techniques with which they were identified (metabolite levels in all the samples are found in Supplementary Tables S1–S5). Out of the 21 fatty acids detected, 20 decreased during fermentation (Figure 2A). In most cases, the decrease started in the first fermentation time measured, i.e., 12 h. Only one fatty acid, acid lignoceric, increased as the fermentation time progressed, with its increase being more pronounced in raw milk kefir than in the pasteurized product in which its increment was very slight. Regarding volatile compounds, some alcohols (ethanol and/or alkyl derivatives of other alcohols like propanol, butanol, hexanol, or heptanol) appeared during the fermentation as a result of the yeast metabolism, as well as the major products of bacterial fermentation, i.e., acetic and lactic acid and/or their derivatives after reacting with ethanol (namely ethyl lactate and ethyl acetate) (Figure 2B). Other compounds including alkanes, ketones, aldehydes, organic acids, and their ester derivatives were also found. For most cases, the increase was more pronounced in the raw milk kefir than in pasteurized milk kefir, although there were exceptions as in the case of 2,3-butanedione. Figure 2C–E show the evolution of non-volatile molecules detected by LC-QToF-MS and GC-ToF-MS either using commercial standards (Figure 2D) or by searching the mass spectra in libraries (Figure 2E). Almost all amino acids detected clearly increased with fermentation, which is indicative of their release due to protein degradation by microbial proteases. Some sugars and their derivatives or intermediates, like sucrose, glucose, or glucose-6-phosphate, decreased, as well as lactose, as expected. This was accompanied by the increase in lactic acid as a result of lactic fermentation, and its derivative lactamide. Also, some molecules such as GABA, shikimic acid, dehydroabietic acid, and tyramine were also augmented as the fermentation progressed in both types of milk.

**Figure 3:**
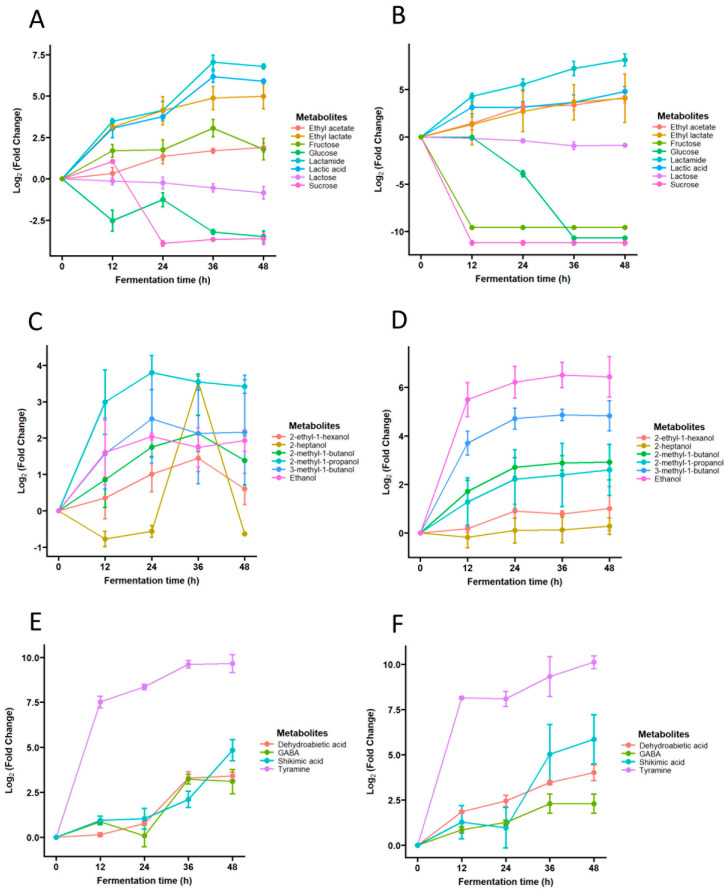
Time-course evolution of selected metabolites in raw (panels **A**,**C**,**E**) and pasteurized goat milk kefir (panels **B**,**D**,**F**).

The evolution of some of the most relevant metabolites of lactic-alcoholic fermentation that occurred in our kefir samples is shown in Figure 3. Figure 3A,B show the changes in electron donors and final acceptors (i.e., sugars, lactic/acetic acid and/or derivatives), in which it is appreciated how sucrose, lactose, and glucose decrease as lactic and acetic acid, as well as how their ethyl esters, together with lactamide, increase. Figure 3C,D show the time-course appearance of alcohols due to alcoholic fermentation caused by yeasts. It can be appreciated that the major alcohol found in kefir, i.e., ethanol, undergoes higher changes in pasteurized milk as the fermentation progresses because of the lower level in the pasteurized non-fermented milk compared to raw unfermented milk. Finally, Figure 3E,F show the evolution of some key metabolites participating in neurotransmission or other processes. Of these, tyramine shows the most pronounced change, as it increased ca. 8-fold in both raw and pasteurized milk after 12 h of fermentation.

### 3.2. NIRS Analysis of Goat Milk Kefir

We also used the NIR spectroscopy technique for characterizing the goat milk kefir and the discrimination between the studied variables, i.e., thermal treatment of milk and fermentation time. The NIR spectra obtained corresponded to those typical of dairy products, with two predominant peaks at 1450 and 1930 nm, which correspond to water (Figure S1). These peaks are so intense that they prevent other characteristic molecules from being detected in the NIR spectrum of this type of product with a high water content. However, the PCA loading plots show areas of the spectrum in which molecular bonds frequently found in milk-based products are present, like acetic acid, at 1678 nm, or lactic acid, at 1950 nm (Figure S2).

**Figure 4:**
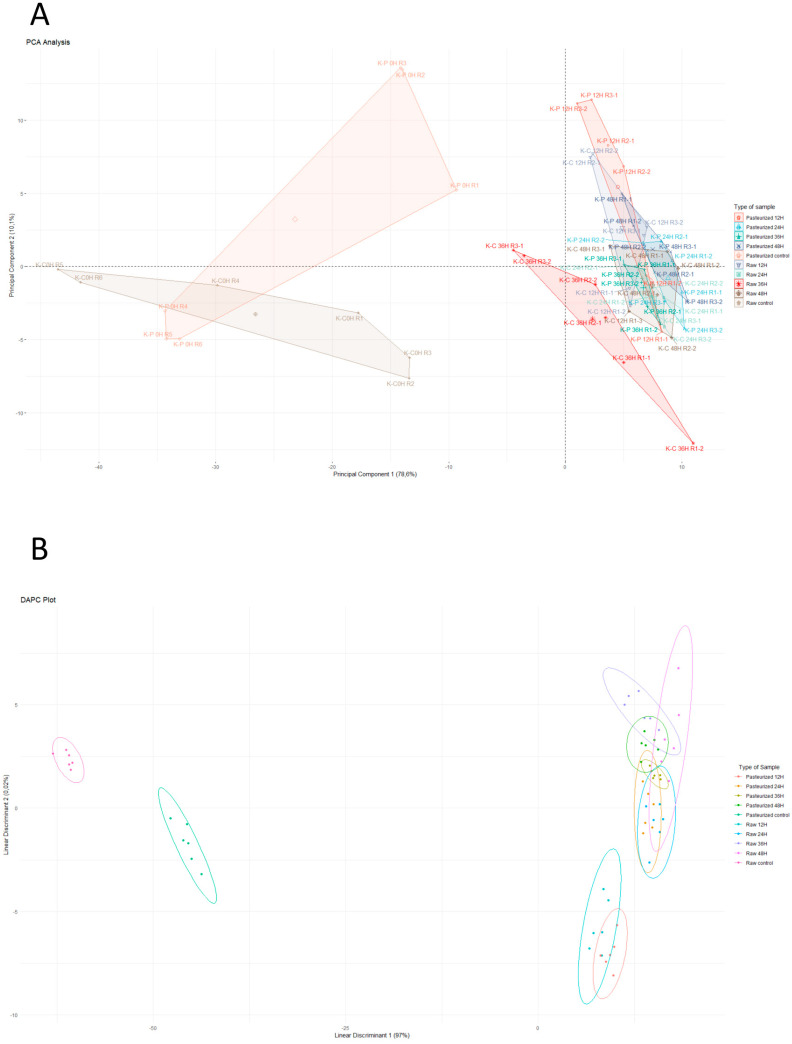
Score plots of two-dimensional principal component analysis (PCA, panel **A**) and discriminant analysis of principal components (DAPC, panel **B**) of NIRS data in goat milk kefir.

Next, we performed a PCA of the NIRS data. The representation of the two first principal components, i.e., PC1 and PC2, explained 88.65% of the variance (Figure 4A). In these analyses, non-fermented samples were clearly differentiated from the fermented ones, although there was not a clear separation between fermentation times. As PCA is a non-supervised analysis, we carried out a discriminant analysis of principal components (DAPC) once we had established the different categories of samples, i.e., according to the type of thermally treated milk and the fermentation time, both using the first two and three principal components (Figure 4B). This analysis showed a clear distinction among fermentation times, but it did not discriminate as precisely between raw and pasteurized milk, as for some time points, the groups corresponding to the same fermentation time were mixed.

### 3.3. Predictive Capacity of Metabolomics and NIRS to Discriminate Kefir Samples

**Figure 5:**
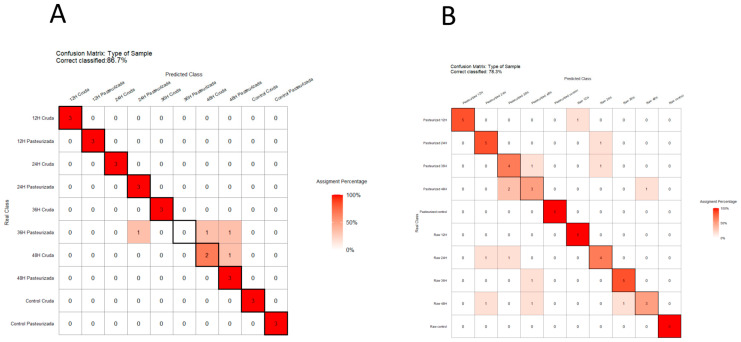
Confusion matrix using LOOCV to evaluate the performance of the model classifying goat milk kefir samples according to fermentation time and thermal treatment of milk. The numbers within each square represent the class assigned by the model. The sample is correctly classified if the real class corresponds with the predicted class (diagonal). (**A**) Confusion matrix for metabolomics data; (**B**) confusion matrix for NIRS data.

Finally, the global data obtained (separately for metabolomics and NIRS) were used to predict the discrimination of kefir samples, both according to the thermal treatment of milk and to fermentation time, using LOOCV model-based confusion matrices (Figure 5). This is a way to calculate the accuracy of the classification of real samples according to predicted categories, serving as cross-validation of the techniques used for the generation of data. Regarding the entire set of metabolomics data, the model obtained 93.3% accuracy (i.e., correctly predicted the classification) for both the type of milk and fermentation time. When combining both factors, i.e., the type of thermal treatment and fermentation time of the milk, the model achieved 86.7% sample classification (Figure 5A). With respect to NIRS data, the model classified the samples with 90% accuracy according to the fermentation time, but the accuracy of classification according to the thermal treatment of milk was lower (68.3%). Considering both variables, sample classification decreased to 78.3% (Figure 5B).

## 4. Discussion

In this work, we have performed the first metabolomic analysis of goat milk kefir by combining different mass spectrometry-based techniques and NIRS for the further differentiation of categories of the final product. Previous studies have focused on the characterization of kefir grains and the microbial composition of kefir [15,20,21], as well as the identification of peptides derived from microbial digestion [9,10,11,12,13,14], but there is limited information on the metabolic changes that occur during milk fermentation by kefir grains. Although metabolomics has been widely applied to characterize other dairy products, like cheese or yogurt [19,22,23,24,25,26], to our knowledge, most of the studies on the metabolomic characterization of kefir published so far, either at a global or targeted level, have been carried out using cow milk [20,21,27,28]. Those involving goat milk kefir have been restricted to a limited group of compounds, mainly volatiles [29]. Therefore, our work provides the first insight into broader changes in the metabolite profiles of goat milk kefir, considering the fermentation time and different thermal treatments as factors affecting such variations.

Our metabolomic analysis identified 105 unique metabolites using different analytical techniques, revealing the complex biochemical composition of goat milk kefir. Additionally, the difference between raw and pasteurized milk in the fermentation process, as evidenced by changes in 27 metabolites, suggests that milk treatment prior to fermentation can influence the metabolomic profile of kefir. The ability of NIRS to distinguish between types of milk and fermentation times further demonstrates the potential of this technique in characterizing fermented foods.

Comparing our findings with existing literature reveals both consistencies and novelties in the metabolomic profile of goat milk kefir. Previous studies have similarly reported the dynamic changes in metabolites during kefir fermentation, reflecting the metabolic activities of the microbial community. However, our study extends the understanding by providing a detailed comparison between raw and pasteurized milk, highlighting how initial milk treatment can influence the fermentation outcome. This aspect has been less explored in previous research, offering a new perspective on the production of goat milk kefir. Microbes present in milk or those responsible for fermentation cause the appearance of volatile compounds conferring the characteristic flavor to dairy products [30,31]. Therefore, it is expected that killing microorganisms by pasteurization will alter the metabolite profile.

By applying a comprehensive metabolomic analysis covering non-polar to polar and volatile compounds, we found the major categories of metabolites described in cow milk kefir and in other dairy products: fatty acids; amino acids, sugars, and other polar molecules; volatiles (alcohols, ketones, aldehydes, and esters); organic acids; and other compounds, including polyols. Regarding fatty acids, these compounds are commonly found in fermented milk including yogurt and kefir of different species, as well as in cheese and butter [29,32]. Out of the 21 fatty acids identified in our work, 20 decreased during fermentation in both types of milk, as also described recently in cow milk kefir [15]. We also found that fermentation resulted in the release of single amino acids, as also described in cow milk kefir [15], which is clearly related to the protease activity of microbes present in the kefir grains. The gradual increase in the amino acid concentrations as the fermentation time progresses is in accordance with our previous findings of peptide release by a peptidomic approach [13]. Similar changes in the concentrations of sugars, alcohols, ketones, aldehydes, and esters have been described in cow milk kefir [15,28], where some concentrations increased and others decreased as the fermentation progressed. Interestingly, some compounds are exclusive of kefir fermentation due to the presence of yeast in the grains, such as ethyl acetate or 3-methyl-1-butanol, as also described for cow milk kefir [28]. During our research, we also observed a decrease in organic acid concentrations, which was in accordance with that described by Bourrie and colleagues [28].

Other compounds identified in kefir have a described bioactive profile. For instance, shikimic acid possesses antimicrobial activity against *Staphylococcus aureus* [33]. GABA is a neurotransmitter that has also been reported in cheese [25], with positive effects on insomnia, depression, the immune system, and blood pressure [34]. Dehydroabietic acid, on the other hand, is a diterpenoid that increases up to 10-fold after 36 h of goat milk fermentation by kefir grains. This compound and its derivatives exhibit a wide range of bioactive properties, including antiviral, antitumor, wound regeneration, antimicrobial, or gastric protection [35]. Tyramine is a monoamine compound derived from the amino acid tyrosine. In foods, it is often produced by the decarboxylation of tyrosine during fermentation or decay. Fermented dairy products contain considerable amounts of this molecule. A large dietary intake of tyramine can increase blood pressure, so people taking monoamine oxidase inhibitors are recommended not to abuse dairy products to avoid possible hypertensive crises [36,37].

NIRS has been widely used for the characterization of foods, with multiple applications, including the differentiation of products, maturation, or the detection of fraudulent mixtures [38,39,40]. In dairy products, the use of NIRS is also habitual due to its ease and cheapness compared to other massive analysis platforms [41,42,43,44]. In the present work, we have carried out a characterization of the kefir samples aiming at establishing the differences between the two thermal treatments or fermentation times. The NIR spectra were able to distinguish the fermentation time variable with high accuracy, but the precision was lower when discriminating between raw and pasteurized milk. The NIRS data could also be integrated with metabolomics data as the peak changes detected in the region between 900 and 1100 nm and in the large peak at 1940 nm are associated with absorption changes mostly caused by N–H bonds. They may be related to the proteolytic activity of the microorganisms. This is supported by the metabolomics results we obtained, where an increase in free amino acids was observed as the fermentation time progressed, as already described previously at the peptidomic level [13]. The changes observed in the final region of the spectrum between 2000 and 2400 nm are likely caused by C–H bonds mostly attributed to lipids, which is in concordance with the decrease in the concentrations of almost all fatty acid species detected over the fermentation time.

### Limitations and Future Research

While our study provides valuable insights into the metabolomic profile of goat milk kefir, limitations exist, such as the need for validation of identified metabolites through targeted analysis. Future research should also explore the bioavailability and health effects of identified metabolites in clinical trials. Additionally, investigating the impact of fermentation variables, such as kefir grain composition and the fermentation environment, could further elucidate the factors influencing the metabolomic profile of kefir.

## 5. Conclusions

This study represents the most comprehensive metabolomic analysis of goat milk kefir so far, revealing the intricate changes in metabolites during fermentation and the impact of milk treatment. Metabolomics achieves better classification results, but this requires simultaneous analysis of samples by several complex, high-cost, and time-consuming analytical techniques. However, models obtained from NIRS data have a similar or lower classification capacity, although the analysis of samples by this technique is very fast, cheap, and simple, which facilitates its implementation as a quality control tool. Our findings underscore the potential of goat milk kefir as a functional food, contributing to our understanding of its nutritional and therapeutic properties. By elucidating the metabolomic complexity of kefir, this research paves the way for future studies aimed at optimizing the health benefits of fermented foods. The identified metabolites, including significant changes in fatty acids and volatile and non-volatile compounds, have implications for the nutritional value and sensory properties of goat milk kefir. The differences observed between raw and pasteurized milk kefir suggest that selecting the appropriate milk treatment could tailor the health benefits and flavor profile of the final product. These findings contribute to the growing body of knowledge on fermented foods, supporting the development of functional foods that cater to specific health and dietary needs.

## Figures and Tables

**Table 1:** Metabolites detected in goat milk kefir with all the metabolomics techniques.

Technique	Metabolites Detected	Name
GC-FID	21	Arachidic acid, behenic acid, capric acid, caprylic acid, eicosatrienoic acid, elaidic acid, estearic acid, gadolenic acid, lauric acid, lignoceric acid, linoleic acid, linoleidic acid, linolenic acid, margaric acid, myristic acid, myristoleic acid, oleic acid, palmitic acid, palmitoleic acid, palmitoleic1 acid, pentadecanoic acid
UHPLC-MS (QToF)	15	Acetylcarnitine, alanine, butyric acid, creatine, creatinine, Glycerophosphocholine, hippuric acid, hidroxybutyric acid, isoleucine, panthotenic acid, fenilalanine, tyrosine, proline, phosphocoline, valine
GC-MS (QqQ)	26	2-methyl-1-butanol,2-methyl-1-propanol, 3-methyl-1-butanol, 2,3-butanodione, 2,4-dimethylheptane, 2-ethyl-1-hexanol, 2-heptanol, 2-heptanone, 3-methyl-2-butanone, 4-ethyl octane, 4-methyl decane, 4-methyl octane, ethanol, ethyl acetate, vynil acetate, acetoine, acetic acid, methyl benzoate, methyl butanoate, camphene, glycol methacrylate, hexanal, mehtyl hexanoate, ethyl lactate, nonanal, valeraldehyde
GC-MS (Tof)	32	4-hidroxyproline, aspartic acid, citric acid, fumaric acid, galacturonic acid, glutamic acid, malic acid, oxalic acid, shikimic acid, succinic acid, alanine, phenylalanine, fructose, GABA, glycerol, glycine, glucose, glucose-6-phosphate, glutamine, isoleucine, leucine, lysine, maltose, methionine, proline, sucrose, serine, tyrosine, threonine, valine, xylitol, β-alanine
	16	4-hidroxyphenillactic acid, dehidroabietic acid, lactic acid, celobiose, creatine, ethanolamide, phosphate, fucose, lactamide, lactose, myo-inositol, ornithine, tyramine, urea, β-gentobiose, β-mannosylglycerate

## Data Availability

Data are contained within the article and Supplementary Materials.

## Supplementary Materials

The following supporting information can be downloaded at: https://www.mdpi.com/article/10.3390/biom14070816/s1, Figure S1: NIRS spectra of goat milk kefir. (A) Raw milk kefir; (B) Pasteurized milk kefir; Figure S2: Principal component analysis (PCA) loading plots of the three first principal components of goat milk kefir; Table S1: Metabolite levels in kefir samples identified by GC-FID; Table S2: Metabolite levels in kefir samples identified by GC-QqQ-MS; Table S3: Metabolite levels in kefir samples identified by GC-MS-ToF using spectral libraries; Table S4: Metabolite levels in kefir samples identified by GC-MS-ToF using standards; Table S5: Metabolite levels in kefir samples identified by UHPLC-QToF-MS.
