# Supplementary material for: Combined Metabolomic and NIRS Analyses Reveal Biochemical and Metabolite Changes in Goat Milk Kefir under Different Heat Treatments and Fermentation Times
Source: Biomolecules. 2024 Jul 9;14(7):816. doi: 10.3390/biom14070816 (PMC11274602; doi:10.3390/biom14070816)
Supplement: Supplementary file 1 [file biomolecules-14-00816-s001.zip › biomolecules-3078042-supplementary/Supplementary figures.pdf]

SUPPLEMENTARY FIGURES

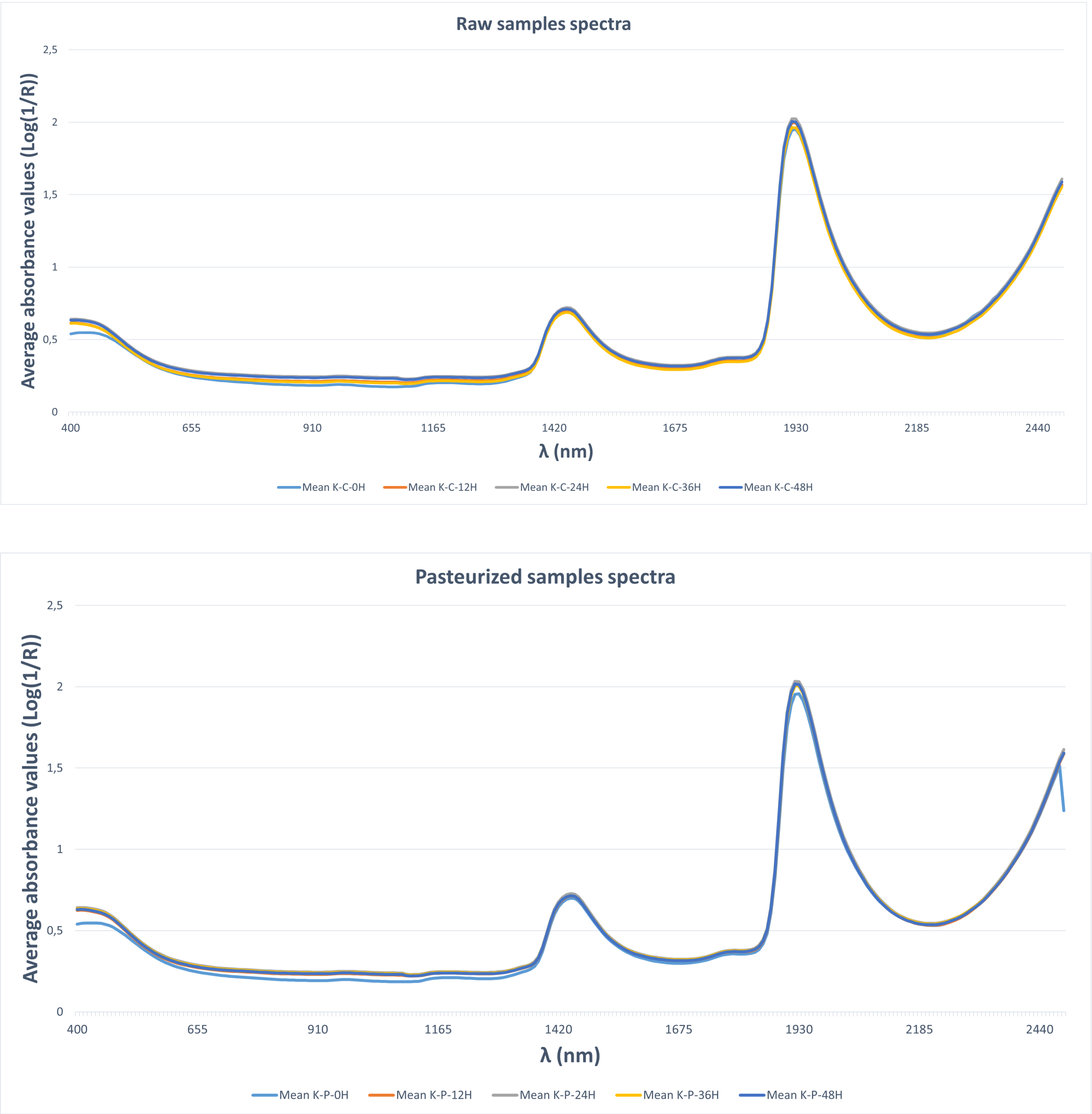

Figure S1. NIRS spectra of goat milk kefir. A) Raw milk kefir; B) Pasteurized milk kefir.

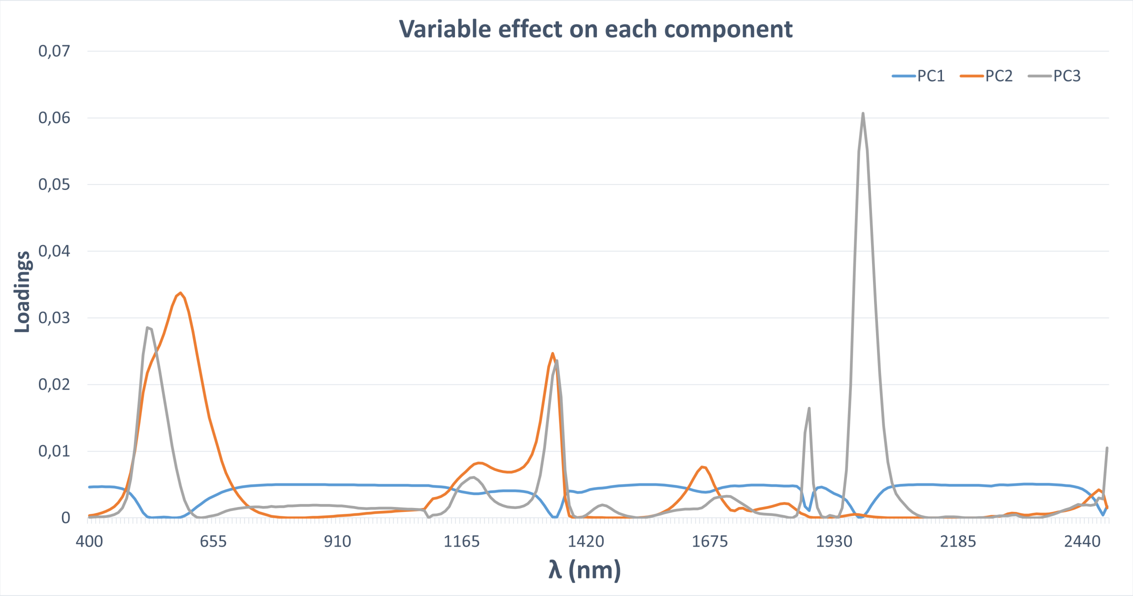

Figure S2. Principal component analysis (PCA) loading plots of the three first principal components of goat milk kefir.
